# Assessing Monkeypox Virus Prevalence in Small Mammals at the Human–Animal Interface in the Democratic Republic of the Congo

**DOI:** 10.3390/v9100283

**Published:** 2017-10-03

**Authors:** Jeffrey B. Doty, Jean M. Malekani, Lem’s N. Kalemba, William T. Stanley, Benjamin P. Monroe, Yoshinori U. Nakazawa, Matthew R. Mauldin, Trésor L. Bakambana, Tobit Liyandja Dja Liyandja, Zachary H. Braden, Ryan M. Wallace, Divin V. Malekani, Andrea M. McCollum, Nadia Gallardo-Romero, Ashley Kondas, A. Townsend Peterson, Jorge E. Osorio, Tonie E. Rocke, Kevin L. Karem, Ginny L. Emerson, Darin S. Carroll

**Affiliations:** 1 U.S. Centers for Disease Control and Prevention, Poxvirus and Rabies Branch, 1600 Clifton Rd. NE, Atlanta, GA 30333, USA; bmonroe@cdc.gov (B.P.M.); ynakazawa@cdc.gov (Y.U.N.); mmauldin@cdc.gov (M.R.M.); zbraden@cdc.gov (Z.H.B.); euk5@cdc.gov (R.M.W.); azv4@cdc.gov (A.M.M.); hfa5@cdc.gov (N.G.-R.); akondas@cdc.gov (A.K.); kkarem@cdc.gov (K.L.K.); gemerson@cdc.gov (G.L.E.); dcarroll@cdc.gov (D.S.C.); 2 University of Kinshasa, Department of Biology, P.O. Box 218 Kinshasa XI, Democratic Republic of the Congo; jean.malekani@unikin.ac.cd (J.M.M.); lemskalemba@yahoo.com (L.N.K.); tresluemba@yahoo.fr (T.L.B.); tobitliyandja@yahoo.fr (T.L.D.L.); divin.malekani@unikin.ac.cd (D.V.M.); 3 Field Museum of Natural History, 1400 S. Lake Shore Dr., Chicago, IL 60605, USA; wstanley@fieldmuseum.org; 4 Biodiversity Institute, University of Kansas, 1345 Jayhawk Blvd., Lawrence, KS 66045, USA; town@ku.edu; 5 University of Wisconsin, School of Veterinary Medicine, 2015 Linden Dr., Madison, WI 53706, USA; osorio@svm.vetmed.wisc.edu; 6 U.S. Geological Survey, National Wildlife Health Center, 6006 Schroeder Rd., Madison, WI 53711, USA; trocke@usgs.gov

**Keywords:** habitat analysis, human–animal interface, monkeypox, orthopoxviruses, serology, small mammals, wildlife

## Abstract

During 2012, 2013 and 2015, we collected small mammals within 25 km of the town of Boende in Tshuapa Province, the Democratic Republic of the Congo. The prevalence of monkeypox virus (MPXV) in this area is unknown; however, cases of human infection were previously confirmed near these collection sites. Samples were collected from 353 mammals (rodents, shrews, pangolins, elephant shrews, a potamogale, and a hyrax). Some rodents and shrews were captured from houses where human monkeypox cases have recently been identified, but most were trapped in forests and agricultural areas near villages. Real-time PCR and ELISA were used to assess evidence of MPXV infection and other *Orthopoxvirus* (OPXV) infections in these small mammals. Seven (2.0%) of these animal samples were found to be anti-orthopoxvirus immunoglobulin G (IgG) antibody positive (six rodents: two *Funisciurus* spp.; one *Graphiurus lorraineus*; one *Cricetomys emini*; one *Heliosciurus* sp.; one *Oenomys hypoxanthus*, and one elephant shrew *Petrodromus tetradactylus*); no individuals were found positive in PCR-based assays. These results suggest that a variety of animals can be infected with OPXVs, and that epidemiology studies and educational campaigns should focus on animals that people are regularly contacting, including larger rodents used as protein sources.

## 1. Introduction

Monkeypox virus (MPXV), a member of the species *Monkeypox virus*, is a zoonotic virus endemic to Western and Central Africa, and is a member of the genus *Orthopoxvirus* (OPXV). MPXV was first detected and isolated from primates at the Statens Seruminstitut in Copenhagen, Denmark, in 1958, and was later observed in several zoos and laboratory primate colonies [1,2]. During the smallpox (*Variola virus*) eradication campaign of the 1970s, monkeypox (MPX) was recognized as a human disease in Western and Central Africa. After smallpox eradication, MPXV became the most pathogenic OPXV to humans. Based on genetic, geographic, and phenotypic (pathogenicity in humans and animal models) variation, MPXV is divided into two distinct clades, West African and Congo Basin, with viruses assigned to the latter being the most virulent [3].

Breman et al. [4] conducted a serosurvey of non-human primates in West Africa (Côte d’Ivoire, Mali and the Upper Volta Region), and found anti-OXPV antibodies in primates of the genera *Cercopithecus* (8%, *n* = 170) and *Colobus* (6%, *n* = 16). Since that report, several studies have attempted to identify the reservoir or natural hosts of MPXV; the results suggested that small mammals played a role in the maintenance and circulation of the virus in nature. Khodakevich et al. [5] conducted an extensive, multi-year ecological survey of rodents and non-human primates at four locations in the Democratic Republic of the Congo (DRC). They reported an anti-OPXV seroprevalence of 23.7% (*n* = 659) in rope squirrels (*Funisciurus* spp.), 14.9% (*n* = 101) in sun squirrels (*Heliosciurus* spp.), and 7.8% (*n* = 12) in non-human primates (primarily *Cercopithecus ascanius*). Hutin et al. [6] also found high OXPV seroprevalence in two species of rope squirrels, 50% (*n* = 4) in *F. anerythrus* and 38.9% (*n* = 18) in *F. congicus*. They also showed serologic evidence of previous OPXV infection in giant pouched rats (*Cricetomys emini*; 15.8%, *n* = 19), elephant shrews (*Petrodromus tetradactylus*; 33.3%, *n* = 3), sun squirrels (50%, *n* = 2), and domestic pigs (*Sus scrofa*; 100%, *n* = 1). In 2013, a MPXV outbreak investigation in Bokungu, Tshuapa Province, DRC, reported anti-OPXV antibodies in two of three (66.7%) rope squirrels collected along with 65 other terrestrial small mammals (rodents and shrews) [7].

Some of the serologic results from previous publications suggest MPXV-specific antibodies were detected in samples collected from small mammals [4,5]. However, the assays used at the time these investigations were reported (radioimmunoassay absorption and indirect immunofluorescence), were not designed to discriminate among the diversity of the genus as it is currently recognized, and this raises questions about the utility of these assays as compared to the OPXV generic immunoglobulin G (IgG) ELISA.

Following the importation of MPXV to the United States through the pet trade in 2003, an ecological survey was conducted in Ghana at the source of the animals imported into the US [8]. Reynolds et al. [8] reported antibody positive dormice (*Graphiurus* sp., 10%, *n* = 90), giant pouched rats (2.6%, *n* = 38), rope squirrels (40%, *n* = 5), and sun squirrels (14.2%, *n* = 7). They also detected OPXV DNA via real-time PCR in dormice (6%, *n* = 100), giant pouched rats (5%, *n* = 40), and unstriped ground squirrels (*Xerus* sp., 100%, *n* = 1).

Despite this serologic and PCR evidence, MPXV has been isolated from wild animals on only two occasions, once from a rope squirrel (*F. anerythrus*) and once from a sooty mangabey (*Cercocebus atys*) [9,10]. The squirrel was killed by a local hunter in the DRC and was obviously ill with pox-like lesions present, while the sooty mangabey was found dead with pox-like lesions in Tai National Park, Côte d’Ivoire. Despite the identification of several species as potential hosts/reservoirs, the rarity of MPXV isolation from wildlife presents a barrier to understanding the transmission cycle of the virus and its maintenance in nature. Contact between humans and infected wildlife is believed to be the primary mode of transmission of MPXV into human populations [5,6]. Therefore, surveys for MPXV exposure in sylvatic species in endemic areas remain one of the best approaches to identifying zoonotic sources of infection and understanding the sylvatic cycle of this virus.

The vast majority of MPX cases are reported from Central Africa, and the DRC in particular, which has an estimated incidence rate of 5.53 cases per 10,000 people [11]. MPX is endemic in the Tshuapa Province, DRC, where an average of 661 suspected human cases were reported per year from 2011 to 2014 (data provided by Ministry of Health, Kinshasa, DRC). In this study, we collected samples from sylvatic animals to assess MPXV prevalence in small mammal communities at the human–animal interface at several locations near Boende, the capital of Tshuapa Province. Additionally, genetic diversity of seropositive squirrels was examined through phylogenetic analyses, and potential associations between seropositive animals and major vegetation types were investigated.

## 2. Materials and Methods

### 2.1. Study Area

**Figure 1:**
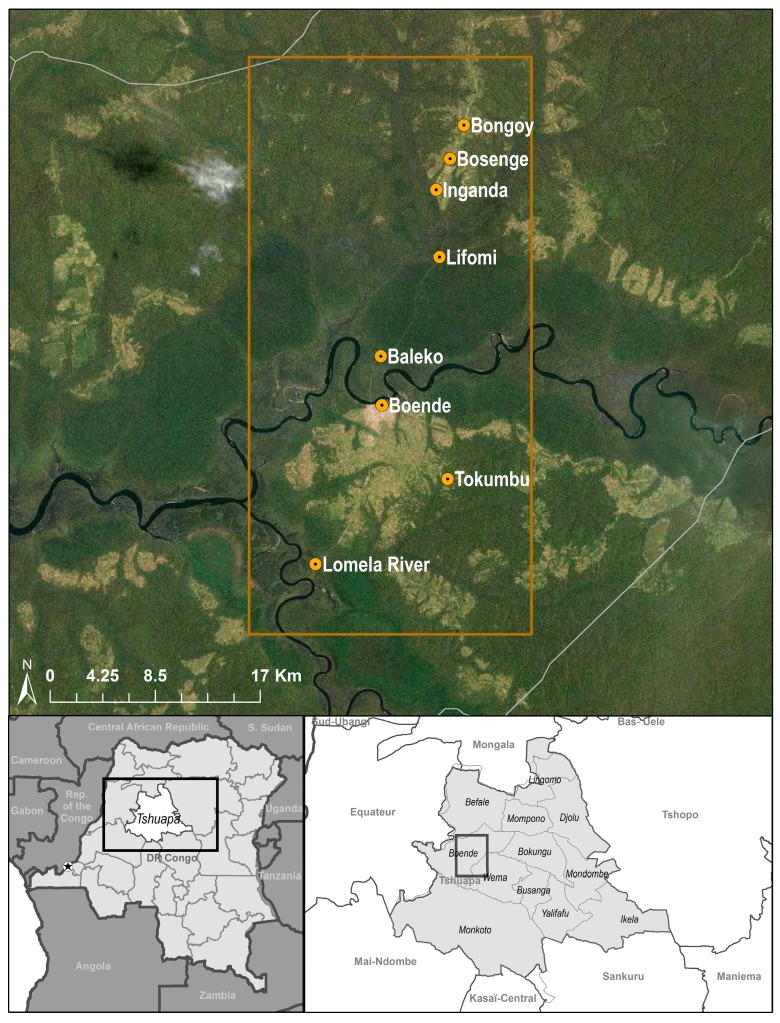
The location of the collection sites is indicated by the orange circles in the top figure. The orange rectangle indicates the area used to extract principal component values. Bottom figures indicate the location of Tshuapa Province (**left**) and the 12 health zones within the province (**right**).

The Boende Health Zone is one of 12 health zones within Tshuapa Province, DRC (Figure 1). It has an area of 10,275 km^2^ and an estimated population of 249,588 people (2014 Tshuapa demographic data), with a population density of 24 persons per km^2^. The vegetation of this health zone is typical of the lowland Congo Basin rainforest. A total of eight localities (Baleko, Boende, Bongoy, Bosenge, Inganda, Lifomi, Lomela, and Tokumbu) within 25 km of the town of Boende were sampled. All sample collection sessions were conducted between May and July of 2012, 2013, and 2015. These sites included seasonally flooded primary forest, secondary forest, agricultural areas, and villages. There are two dry seasons, one from January to early March, and another from June to early September; the remaining time periods are rainy seasons. The area receives an average of 210.9 cm of rain per year and daily temperatures average between 24 °C and 30 °C year-round (data provided by Ministry of Health, Province of Tshuapa, DRC).

Some structures within the health zone were built during colonial times and are made with concrete floors and walls, but these constructions typically are limited to more developed towns, health centers, and religious facilities. Houses in rural areas within Tshuapa are typically constructed of locally available materials such as mud brick, wood, and palm thatch with corrugated tin roofs on some houses [12]. Like housing materials, many other needs (e.g., food sources, cooking oils, baskets, firewood) are commonly met through harvesting forest products. Wild game, regularly referred to as bushmeat, is a common source of protein in many parts of Africa, including the forested areas of DRC [13,14]. Many villagers set traps and snares to hunt for small-to-medium-sized mammals (squirrels and other rodents, elephant shrews, pangolins, etc.) [15]. Animals found sick or dead in the forest are also collected for consumption or for distribution through a market [16].

### 2.2. Animal Capture and Sample Collection

Animals were captured with H.B. Sherman live traps (H.B. Sherman Traps Inc., Tallahasse, FL, USA), snap traps, and pitfall traps; additionally, some animals were purchased from local hunters. In areas with natural or disturbed vegetation, a mixture of Sherman and snap traps were arranged in transects of 50 traps each. Pitfall traps were constructed with 11 buckets, spaced 5 m apart, and plastic sheeting, slightly buried to create a barrier for the animals to run along before falling into the buckets. Traps were kept in the same location for four to eight nights to increase diversity of captured animals. Traps were also placed in and around the houses of suspected human MPX cases and other houses in nearby neighborhoods.

Captured animals were transported to a central processing site. Isoflurane, USP (Phoenix St. Joseph, MO, USA) was used to anesthetize live animals prior to blood collection via cardiac stick. Animals were euthanized by exsanguination followed by cervical dislocation. Standard measurements, relative age (juvenile or adult), and reproductive characteristics were recorded for each animal. Animals were assessed for the presence of lesions and obvious wounds. Samples of liver (two samples), lung, spleen, heart, kidney, and brain were collected and placed into 2 mL cryotubes and stored in liquid nitrogen. If lesions were observed, swabs and lesion material were collected; in 2015, oral swabs were also collected from each animal. A dried blood sample was also collected on Nobuto filter paper (Advantec, Tokyo, Japan). All specimen material (carcasses and skulls) and one sample of liver per animal were deposited in the Field Museum of Natural History (Chicago, IL, USA); blood samples and other tissue samples were sent to Centers for Disease Control and Prevention (CDC) (Atlanta, GA, USA) for diagnostic testing. This work was conducted under CDC Institutional Animal Care and Use Committee (IACUC) approved protocols (2344RUBMULXZ (8 February 2012) and 2660GALMULX (12 March 2015)).

### 2.3. Laboratory Diagnostics

Serum samples and blood recovered from Nobuto filter paper (Advantec) were assessed by modified enzyme-linked immunosorbent assay (ELISA) for the presence of anti-OPXV IgG antibodies [17,18] in sample dilutions of 1:100, 1:200, and 1:400. Crude *Vaccinia virus* (Western Reserve) at a concentration of 0.01 µg/well diluted in carbonate buffer was used for coating half of each microtiter plate. The other half of the plate was coated with an equal concentration of BSC-40 cell lysate (CDC Core Facility, Atlanta, GA, USA). The average of the OD values from the duplicates of a sample in the virus half of the plate, minus the average plus two standard deviations of the duplicates from the corresponding sample in the cell lysate half of the plate, was used to generate a cut-off value (COV). An animal was confirmed positive for the presence of anti-OPXV antibodies if the sample OD value was above the COV in at least two consecutive dilutions (1:100 and 1:200).

DNA was isolated from liver samples with the Qiagen M48 robot and the Qiagen MagAttract DNA Mini M48 Kit (Qiagen Inc., Valencia, CA, USA). The presence of viral DNA was assessed using real-time PCR to detect the *E9L* gene of orthopoxviruses as described by Li et al. [19].

### 2.4. DNA Sequencing

Given previously documented associations between squirrels and MPXV, a basic assessment was conducted of the genetic variation and species-level diversity of the squirrels collected during the study. The entire mitochondrial cytochrome b (*Cytb*) gene (1140 bp) was amplified with PCR methods outlined by Edwards and Bradley [20] using primers LGL765 [21] and LGL766 [22] (CDC Core Facility). PCR products were electrophoresed through a 2% agarose gel stained with ethidium bromide (Invitrogen, Grand Island, NY, USA) with a 1 kb ladder. Amplicons of expected size were purified using QIAquick PCR Purification Kit (Qiagen Inc.) as prescribed by the manufacturer. Sequencing reactions were performed using BigDye Terminator v.3.1 Cycle Sequencing Kit (Applied Biosystems, Foster City, CA, USA) with the two PCR primers, as well as MVZ05 [23] and 400R [24] (CDC Core Facility). Cycle sequencing reactions were purified using the DyeEx 2.0 Spin Kit (Qiagen Inc.) and analyzed on an ABI PRISM 3130-Avant Genetic Analyzer (Applied Biosystems).

Sequences were proofed using Geneious v.7.1.7 (Biomatters Ltd.; Auckland, New Zealand) and aligned by hand using MEGA6 software [25]. All sequences generated for this study were deposited into GenBank (Accession numbers MF577066-MF577076). A test for the most appropriate model of molecular evolution, according to the Akaike Information Criterion (AIC), and between-group mean genetic (p) distances was also conducted using MEGA6. Bayesian inference analyses were preformed using MrBayes 3.2.2 [26,27]. Two analyses were executed, the first included taxa from multiple tribes including Callosciurini, Marmotini, Protoxerini, and Xerini, and utilized *Aplodontia rufa* as the outgroup . Given results of this analysis, the second analysis included 11 sequences, including data from eight squirrels collected during this study and three sequences from squirrels collected during the Bokungu outbreak investigation [7] and reference samples of Protoxerini (GenBank numbers: HQ450776-HQ450779, KJ193379, U59179 and the selected outgroup taxon (*Xerus* (GenBank: DQ010393—based on topology reported in Mercer & Roth [28])). Settings for Bayesian analyses included NST = 2, rates = gamma, 20 million iterations samplefreq = 2000 burnin = 2500, with four chains, and a majority rules consensus tree. Genetic distance calculations were performed in MEGA6 with Kimura 2 parameters [29].

### 2.5. Habitat Analysis

**Figure 2:**
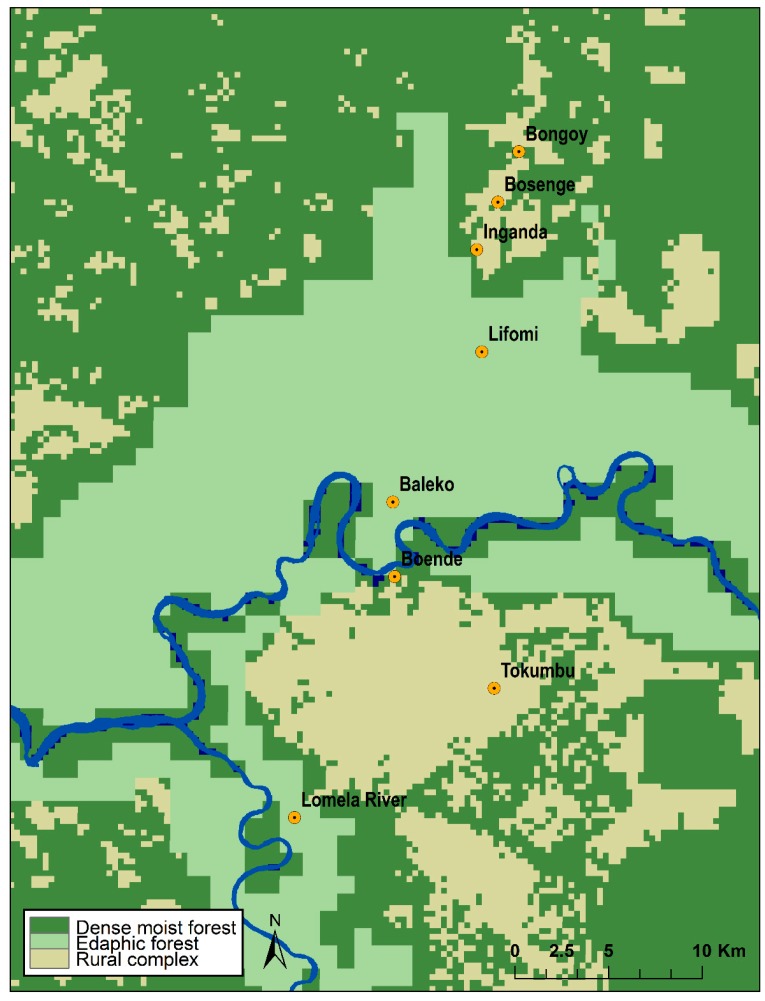
Vegetation types for our study area based on Verhegghen et al. (2012) [36].

To analyze general characteristics of the vegetation surrounding collection sites, we obtained the 16-day maximum value composites of the enhanced vegetation index (EVI) products at 250 m resolution derived from the Moderate Resolution Imaging Spectroradiometer (MODIS), covering five years (2010–2014). These products were obtained from the Land Processes Distributed Active Archive Center (LP DAAC; Sioux Falls, SD, USA; https://lpdaac.usgs.gov/) Vegetation indices are used to monitor photosynthetic activity, health, biomass and structure of vegetation, among other parameters [30]. In total, we obtained 115 composite layers (23 16-day periods per year for five years). Mean EVI values for each 16-day period were obtained by calculating the average of EVI values recorded for a particular period during the five years, resulting in 23 mean EVI layers (one per 16-day period). A 1 km buffer was created around the trapping locations to represent the most likely area of origin of the collected animals; mean EVI values of all pixels within the buffer were calculated using ArcGIS 10.3 (Esri, Redlands, CA, USA) for each of the 16-day periods; the time series was plotted for comparison. Additionally, the 23 layers of mean EVI values for the entire area were used in a principal component analysis in ArcGIS 10.3; principal component values for the pixels within our area of interest (orange rectangle in Figure 1 and Figure 2) and at the collection localities were extracted and used to visually compare their environmental characteristics.

## 3. Results

### 3.1. Animal Capture

In all, 353 animals were captured during 4036 trap nights at eight locations (Table 1). The collected mammals belong to the orders Rodentia (281; 74.9%), Soricomorpha (70; 18.7%), Macroscelidea (16; 4.3%), Pholidota (6; 1.6%), Insectivora (1; 0.3%) and Hyracoidea (1; 0.3%).

Sixteen individuals, including shrews (*Crocidura* spp.) and typical peridomestic rodents (*Rattus* and *Mus*) were captured in and around eight households in Lifomi and Boende (Table 2).

### 3.2. Laboratory Diagnostics

Seven of 353 (2.0%) serum specimens were found to be positive for anti-OPXV IgG antibodies by ELISA (Table 3). Six of the positive samples were collected from rodents (Rodentia): rope squirrels (*Funisciurus* spp., 2/6, 33.3%); an African dormouse (*Graphiurus lorraineus* 1/13, 7.7%; a giant-pouched rat (*Cricetomys emini* 1/9, 11.1%); a sun squirrel (*Heliosciurus* sp., 1/3, 33.3%); a rusty-nosed rat (*Oenomys hypoxanthus*, 1/22, 4.5%); one positive sample was collected from an elephant shrew (order: Macroscelidea, *Petrodromus tetradactylus*, 1/17, 5.9%). All samples from all locations and habitats were found to be negative for the presence of OPXV DNA by PCR.

### 3.3. Phylogenetics

**Figure 3:**
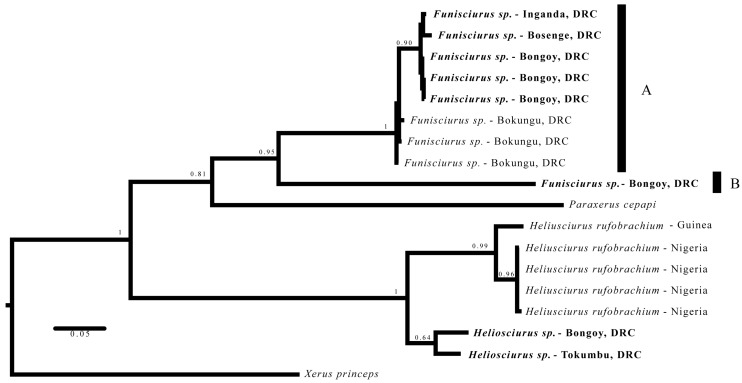
Molecular phylogenetic analysis by Bayesian inference. Majority rules consensus tree generated with MrBayes 3.2.2. Numbers above nodes indicate Bayesian posterior probabilities. Samples with bold font were collected by this study. Letters A and B indicate lineages within the genus *Funisciurus* that warrant further examination, as they are at least 24% divergent from their recovered sister taxon.

Based on monophyletic groupings and high Bayesian posterior probabilities, the phylogenetic analyses (Figure 3) in conjunction with sequence divergence calculations (Table 4) suggested that the collected squirrels belong to two genera representing two species of *Funisciurus* and one species of *Heliosciurus*. Relationships between *Funisciurus*, *Paraxerus*, and *Heliosciurus* were consistent with previously published multigene datasets when missing taxa are considered [28]. One individual of each *Funisciurus* species was found to be antibody positive. The other seropositive animals were identified morphologically.

### 3.4. Habitat Analysis

**Figure 4:**
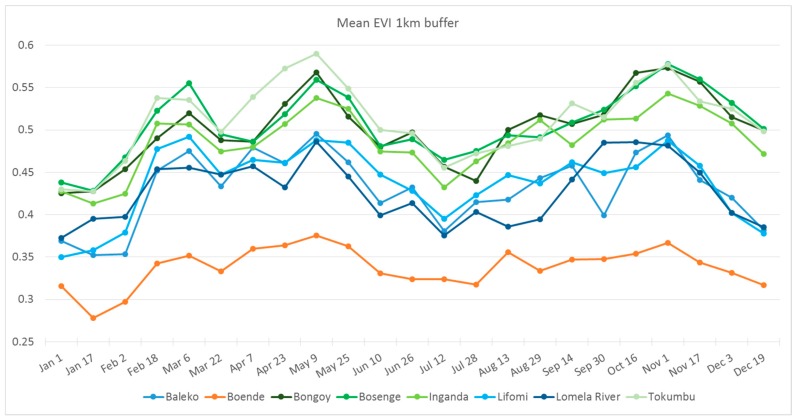
Time series of five-year average enhanced vegetation index (EVI) values within a 1 km buffer from each of the collection localities. Bongoy, Bosenge, Inganda, and Tokumbo showed higher EVI values throughout the year than the rest of the localities.

**Figure 5:**
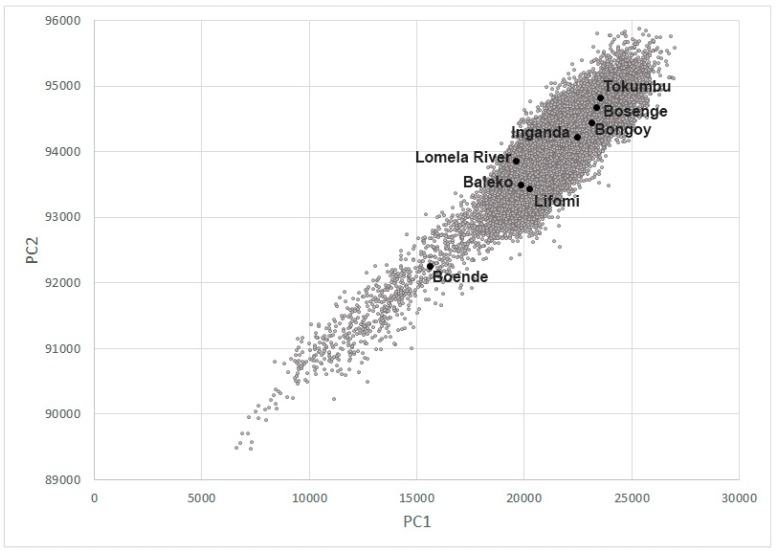
Scatterplot of principle component 1 (PC1) and principle component 2 (PC2) calculated within the study area (gray) and corresponding values for the collection sites (black).

The mean EVI values calculated within the 1 km buffers for Bongoy, Bosenge, Inganda, and Tokumbo are consistently higher than the other localities throughout the year (Figure 4); Baleko, Lifomi, and Lomela River have lower EVI values than localities mentioned above, but higher than those presented by Boende. These differences are also shown when principal component values are plotted (Figure 5), where the four localities with higher EVI values are grouped on the top right part of the plot, the three localities with intermediate EVI values are in the middle of the plot and the dot corresponding to Boende is towards the lower left part of the plot.

## 4. Discussion

The evidence presented here supports previous studies suggesting that squirrels of the genera *Funisciurus* and *Heliosciurus* are involved in the natural cycle of MPXV in DRC [5,6,7,9,31,32]. Rodents of the genera *Cricetomys* and *Graphiurus*, as well as elephant shrews of the genus *Petrodromus*, have also been previously associated with MPXV infections in DRC [6]. The ELISA used in this study cannot distinguish between infections of MPXV and other OPXVs. In this work, the presence of anti-OPXV antibodies was used as an indicator of previous MPXV infection since there are no other known OPXVs in this area. However, it is possible that our positive serology results could represent infections with MPXV or other cryptic OPXVs circulating in these animal populations; this could potentially include OPXVs that are non-pathogenic to humans and therefore go undetected.

Through informal discussions with local residents and interviews associated with MPX outbreaks in the region, we learned that squirrels are hunted for food by both adult men and young children. Nolen et al. [7] describes a case in which children brought home a squirrel and played with it prior to consuming the meat, and that one of the children became ill with MPX five days after the animal died. Stories like this suggest rope squirrels could be involved in the primary transmission of MPXV to humans. Despite these observations, Nolen et al. [7] found no significant association between contact with squirrels and human MPXV infections during that outbreak. Making these types of statistical correlations can be difficult in this area, since the majority of the population relies on bushmeat as a source of protein and many report recent contact with squirrels [7,16], yet only a portion of these individuals had evidence of MPXV exposure. Giant-pouched rats and elephant shrews are also popular food sources for the local population [33] and could be potential sources of MPXV infection for human communities.

Genetic data indicated two species of rope squirrels were sampled in this study. Nolen et al. [7] examined three specimens of *Funisciurus* in their MPXV outbreak investigation from the adjacent Bokungu Health Zone, and we included genetic data from those individuals in our analyses. Three species are listed by Kingdon [34] as occurring in the area: *F. congicus*; *F. anerythrus*, and; *F. pyrropus*. We identified rope squirrel No. 844 as *F. congicus* based upon morphological characteristics; the genetic data support that identification. Based on Bayesian Posterior Probabilities, monophyly, and genetic distance calculations, four of the remaining five rope squirrels are likely *F. congicus* as well. One rope squirrel (No. 1110, anti-OPXV positive) grouped as sister to the *F. congicus* samples, but was more than 24% divergent at the *Cytb* locus based on Kimura 2 parameter calculations. This level of genetic divergence is sufficient for genus-level recognition in small mammal taxa [35], and either represents broad genetic diversity within the genus *Funisciurus* or this specimen could represent another cryptic genus; all of these specimens were identified as the same species in the field based upon pelage color and external measurements. The *Heliosciurus* sp. sequences generated in this study group with *Heliosciurus rufobrachium* sequences from West Africa (Nigeria and Guinea). However, the genetic distance between our sequences and those obtained through GenBank show 11.39% divergence and could represent samples from a different species or distinct geographic sub-species. Unfortunately, due to the lack of additional genetic information available on GenBank, we are currently unable to make direct comparisons with previously published sequences and must therefore rely upon the analysis detailed above. This limits our ability to identify these squirrels definitively beyond genus level and should be examined more closely utilizing both morphological and molecular data in the context of a larger dataset.

We did not find any animals with evidence of current or past MPXV infection in or around houses, which suggests that human MPXV infections are likely not coming from animals in the home. Despite the small samples size, this is further supported by the fact that usual household pests (*Rattus*, *Mus*, and *Crocidura*) have not been found to have any evidence of infection in sylvatic settings. Nolen et al. [7] found similar results in the adjacent Bokungu Health Zone of Tshuapa Province, DRC, during an investigation of a human MPX outbreak in 2013.

Three seropositive animals were captured near Tokumbo (*Heliosciurus*, *Graphiurus*, and *Oenomys*), two were captured near Bongoy (*Funisciurus* sp. and *Petrodromus*), one near the village of Inganda (*F. congicus*), and one was captured in Boende (*Cricetomys*). The habitat analyses showed differences between the eight collection localities that resemble those found by Verhegghen et al. [36] between dense moist forest and edaphic forest. The four localities with higher EVI values seem to be surrounded by dense vegetation (dense moist forest). Baleko, Lifomi and Lomela River showed lower EVI values, corresponding to edaphic forest in Verhegghen et al. [36], which is consistent with field observations of these areas being flooded, at least seasonally. Boende represented the only collection site within an area with high anthropogenic disturbance. Antibody-positive animals came from locations with no indication of seasonal flooding (Boende, Bongoy, Inganda and Tokumbo), which suggests that MPXV host(s) might prefer habitats that are at slightly higher elevations and that do not regularly flood.

We found a seropositive individual of one species not previously identified as a potential host for MPXV, the rusty-nosed rat. Given the broad host range of MPXV and orthopoxviruses in general [37], it is not surprising to find potential spillover events into other taxa, which highlights the difficulty in determining the reservoirs of MPXV. Given the large number of mammal species that have been associated with anti-orthopoxvirus IgG and MPXV DNA [4,5,6,8], the most likely scenario is that MPXV is maintained in nature by a group of diverse reservoir taxa, rather than a single species. Additional reservoir species might still be identified by future wildlife surveillance studies, however, the current available information about MPXV infections in small mammals can be used to educate local communities about the risks of collecting, preparing, and consuming meat from these animals.

## Figures and Tables

**Table 1:** Mammals collected by taxonomic order and location, the Democratic Republic of the Congo 2012–2015.

	Baleko	Boende	Bongoy	Bosenge	Inganda	Lifomi	Lomela	Tokumbu	Total
Rodentia	86	29	6	1	6	95	14	25	262
Soricomorpha	33	6				26	2		67
Pholidota						6			6
Macroscelidea			12		4				16
Hyracoidea	1								1
Insectivora		1							1
Total	120	36	18	1	10	127	16	25	353

**Table 2:** Rodents captured in houses in Lifomi and Boende, the Democratic Republic of the Congo 2013. These animals are also included in data from Table 1.

Genus	No. Sampled
*Rattus*	7
*Mus*	6
*Crocidura*	3
Total	16

**Table 3:** Anti-orthopoxvirus immunoglobulin G (IgG)-positive animals by collection year and location, the Democratic Republic of the Congo 2012–2015. Locations in bold represent sites where seropositive animals were collected.

	2012 Localities	2013 Localities	2015 Localities	IgG Positive
*Funisciurus* spp.	None	**Inganda**	**Bongoy**, Bosenge	2/6, 33.3%
*Graphiurus lorraineus*	None	None	Inganda, **Tokumbu**	1/13, 7.7%
*Cricetomys emini*	None	None	**Boende**, Tokumbu	1/9, 11.1%
*Heliosciurus rufobrachium*	None	None	Bongoy, **Tokumbu**	1/3, 33.3%
*Oenomys hypoxanthus*	Baleko	Lifomi	Inganda, **Tokumbu**	1/22, 4.5%
*Petrodromus tetradactylus*	None	Inganda	**Bongoy**	1/17, 5.9%

**Table 4:** Genetic distances as calculated by MEGA6 using the Kimura2 model of molecular evolution for squirrels sampled in Tshuapa Province, the Democratic Republic of the Congo, 2012–2015. FuniA represents *Funisciurus* sequences from clade A on Figure 3, FuniB represents *Funisciurus* sequences from clade B on Figure 3, HelioSamples represents *Heliosciurus* samples sequenced in this study and HelioRefs represents *Heliosciurus* sequences obtained through GenBank.

	FuniA	FuniB	HelioSamples	HelioRefs	Paraxerus
FuniB	24.23%				
HelioSamples	30.22%	33.65%			
HelioRefs	25.62%	28.04%	11.39%		
Paraxerus	29.05%	30.75%	36.38%	30.05%	
Xerus	29.88%	33.43%	33.61%	32.46%	34.15%

## Appendix A

**Table A1:** Total list of specimens collected during this study arranged by order and genus, the Democratic Republic of the Congo, 2012–2015.

Order	Genus	No. Sampled	No. IgG Positive
Rodentia	*Praomys*	102	0
Rodentia	*Graphiurus*	13	1, 7.7%
Rodentia	*Oenomys*	14	1, 7.1%
Rodentia	*Cricetomys*	10	1, 10%
Rodentia	*Funisciurus*	6	2, 33.3%
Rodentia	*Heliosciurus*	3	1, 33.3%
Rodentia	*Lemniscomys*	6	0
Rodentia	Unknown squirrel	1	0
Rodentia	*Rattus*	29	0
Rodentia	*Mus*	21	0
Rodentia	*Dendromus*	1	0
Rodentia	*Deomys*	4	0
Rodentia	*Lophuromys*	23	0
Rodentia	*Hylomyscus*	5	0
Rodentia	*Hybomys*	16	0
Rodentia	*Malacomys*	4	0
Rodentia	*Atherurus*	2	0
Rodentia	*Anomalurus*	1	0
Rodentia	*Stochomys*	1	0
Soricomorpha	*Crocidura*	51	0
Soricomorpha	*Sylvisorex*	13	0
Soricomorpha	*Scutisorex*	3	0
Macroscelidea	*Petrodromus*	16	1, 6.3%
Pholidota	*Manis*	6	0
Insectivora	*Potamogale*	1	0
Hydracoidea	*Dendrohyrax*	1	0
Total		353	7, 2%
